# Carbonic Anhydrases in Mycobacteria: Linking Metabolic Adaptation to Phenotypic Drug Tolerance

**DOI:** 10.1007/s00284-026-05196-3

**Published:** 2026-10-16

**Authors:** Maneesh Kumar, Ashok Aspatwar

**Affiliations:** 1 State VRDL, Department of Microbiology, All India Institute of Medical Sciences, Deoghar, 814152 Jharkhand India; 2 Faculty of Medicine and Health Technology, Tampere University, Tampere, 33520 Finland

## Abstract

Tuberculosis (TB) treatment is increasingly challenged by both antimicrobial resistance (AMR) and phenotypic drug tolerance, the latter representing a reversible physiological state that contributes to persistence and prolonged therapy. While AMR is driven by heritable genetic mutations, drug tolerance arises from reversible physiological adaptations that enable bacterial survival during prolonged antibiotic exposure. Increasing evidence suggests that metabolic remodeling plays a central role in establishing these drug-tolerant states. Carbonic anhydrases (CAs) are zinc-containing metalloenzymes that catalyze the reversible interconversion of carbon dioxide and bicarbonate, thereby maintaining intracellular pH homeostasis and supporting bicarbonate-dependent metabolism. Beyond their established metabolic functions, indirect evidence suggests that CAs may contribute to physiological adaptations associated with bacterial persistence under host-imposed stresses, including hypoxia, nutrient limitation, and acidic environments. Although they are not classical AMR determinants, CAs may influence the metabolic landscape associated with phenotypic drug tolerance. In this review, we examine current evidence supporting the role of CAs in mycobacterial metabolism and propose a conceptual framework linking bicarbonate homeostasis, intracellular pH regulation, and metabolic adaptation to bacterial persistence and drug tolerance. We further discuss the potential of carbonic anhydrase inhibitors (CAIs) as adjunctive agents whose ability to enhance the activity of conventional antitubercular drugs requires experimental validation, together with the value of experimental models, including the *Mycobacterium marinum*–zebrafish system, and computational approaches for validating CA-targeted therapeutic strategies. Overall, this review highlights CAs as candidate metabolic regulators that may influence the physiological state underlying phenotypic drug tolerance. Defining their mechanistic contribution to persistence could facilitate the development of metabolism-based therapeutic strategies that complement existing TB treatment.

## Introduction

Antimicrobial resistance (AMR) in mycobacteria, particularly *Mycobacterium tuberculosis* (Mtb), remains one of the greatest challenges to global tuberculosis (TB) control [1, 2]. Although resistance is primarily driven by chromosomal mutations in drug targets and other well-characterized mechanisms, these genetic alterations alone cannot fully explain the persistence of infection or the prolonged multidrug therapy required for successful treatment [3–5]. Increasing attention has therefore shifted toward phenotypic drug tolerance, a reversible physiological state that enables bacterial populations to survive antibiotic exposure without acquiring stable resistance-conferring mutations [3, 6, 7]. In this context, drug tolerance refers to the reduced susceptibility of a bacterial population to antibiotic killing, whereas persistence refers to the survival of a subpopulation of phenotypically tolerant cells (persisters) during otherwise lethal antibiotic exposure [3, 5]. Drug-tolerant and persister populations may contribute to treatment failure and relapse and may facilitate the subsequent emergence of genetically resistant strains [8, 9].

Accumulating evidence indicates that drug tolerance is closely linked to metabolic adaptation. During infection, Mtb is exposed to diverse host-derived stresses, including hypoxia, nutrient deprivation, acidic pH, oxidative stress, and immune-mediated antimicrobial responses within granulomatous lesions [10–13]. These conditions induce extensive metabolic remodeling, characterized by altered carbon utilization, reduced replication, changes in energy metabolism, and activation of stress-response pathways that collectively enhance bacterial survival under antibiotic pressure [14–17]. Consequently, identifying metabolic regulators that enable adaptation to these hostile environments has become an important priority in the search for strategies to shorten TB treatment and improve therapeutic efficacy.

Carbonic anhydrases (CAs) are zinc-dependent metalloenzymes that catalyze the reversible hydration of carbon dioxide to bicarbonate, thereby maintaining intracellular bicarbonate homeostasis essential for central metabolism [18–26]. Mycobacteria encode three β-class CAs whose molecular properties, structural characteristics, physiological functions, and potential as antimicrobial drug targets have been extensively reviewed [19–24]. Rather than revisiting these established aspects, the present review focuses on the emerging concept that CAs may function as metabolic regulators coordinating bacterial adaptation to environmental stress.

Although direct experimental evidence remains limited, a growing number of studies suggest that pathways involved in bicarbonate homeostasis, pH regulation, carbon metabolism, and energy balance contribute to physiological states associated with bacterial persistence and phenotypic drug tolerance [26–31]. Within this framework, CAs may represent a metabolic link between environmental sensing and adaptive responses associated with survival during antibiotic exposure. Understanding these relationships could provide new insights into the metabolic basis of drug tolerance and identify opportunities for therapeutic intervention.

Recent reviews have comprehensively summarized the molecular biology, structural features, and inhibitor development of mycobacterial CAs. In contrast, the potential role of these enzymes in metabolic adaptation, persistence, and phenotypic drug tolerance has not been critically synthesized. Therefore, the added value of the present review is to integrate established CA biology with emerging evidence on mycobacterial stress adaptation and drug tolerance, while explicitly distinguishing experimentally demonstrated functions from indirect associations and testable hypotheses. Accordingly, this review examines the current evidence linking CAs with bicarbonate homeostasis, metabolic remodeling, and stress adaptation in mycobacteria. We further discuss recent advances in CO_2_-dependent PhoPR signaling, metabolomic studies, and zebrafish infection models that provide new insights into the physiological functions of these enzymes during host adaptation. Finally, we propose an integrated conceptual framework describing how carbonic anhydrase-mediated metabolic regulation may contribute to bacterial persistence and phenotypic drug tolerance, while highlighting key knowledge gaps and future research priorities.

## CAs in Mycobacteria: Molecular and Functional Overview

CAs (EC 4.2.1.1) are metalloenzymes that catalyze the reversible hydration of carbon dioxide (CO_2_) to bicarbonate (HCO_3_⁻) and protons (CO_2_ + H_2_O ⇌ HCO_3_⁻ + H⁺) [25]. This highly conserved reaction maintains intracellular bicarbonate homeostasis required for central metabolism, biosynthesis, and acid-base balance [25, 26]. In mycobacteria, CA activity provides a metabolic link between carbon utilization, pH regulation, and adaptation to changing environmental conditions, processes that are increasingly recognized as important determinants of bacterial survival during infection [20, 26, 30–32].

The Mtb genome encodes three β-class CAs (CanA/Rv1284, CanB/Rv3588c, and CanC/Rv3273) together with one γ-class carbonic anhydrase (Rv3705c) [21, 23]. The molecular, structural, biochemical and inhibitor profiles of these enzymes have been comprehensively reviewed elsewhere and are therefore not discussed in detail here [19–24]. Instead, this review focuses on their collective physiological significance as regulators of bicarbonate homeostasis and metabolic adaptation.

Functionally, CAs supply bicarbonate for numerous carboxylation reactions involved in central carbon metabolism, fatty acid biosynthesis, anaplerotic pathways, and the synthesis of essential cellular metabolites [19, 25, 26, 33, 34]. These metabolic functions are particularly relevant in mycobacteria because lipid metabolism and cell envelope remodeling are closely associated with intracellular survival, persistence, and adaptation to host-derived stress [14, 16, 35, 36]. By sustaining bicarbonate-dependent metabolism, CAs may contribute to metabolic flexibility under fluctuating environmental conditions.

Beyond their biosynthetic roles, CAs may also contribute to physiological adaptation during infection. Their ability to rapidly interconvert CO_2_ and bicarbonate contributes to intracellular pH homeostasis and buffering, functions that may support physiological adaptation when Mtb encounters acidic pH, hypoxia, nutrient deprivation, oxidative stress, and other hostile conditions within macrophages and granulomatous lesions [25, 37, 38]. Although direct evidence linking CA activity to phenotypic drug tolerance is currently lacking, metabolic adaptations associated with persistence and transient antibiotic tolerance include reduced replication, altered energy metabolism, changes in carbon utilization, and physiological responses to acidic and hypoxic stress [10, 11, 16, 32]. CA-mediated maintenance of bicarbonate and pH homeostasis could potentially support some of these adaptive processes, although this relationship remains hypothetical.

Collectively, rather than acting solely as housekeeping enzymes, CAs may contribute to the metabolic network that enables mycobacteria to adapt to environmental stress. This emerging perspective provides the conceptual basis for examining how CA-dependent metabolic regulation may influence persistence, phenotypic drug tolerance, and ultimately treatment outcome, topics explored in the following sections.

## CAs and Bacterial Physiology Under Stress

Mtb is highly adapted to survive within diverse and hostile host environments, where it encounters hypoxia, nutrient limitation, oxidative stress, acidic pH, and immune-mediated antimicrobial pressure [37, 39]. These stressors are particularly pronounced within granulomatous lesions, where the bacterium resides in microenvironments characterized by limited oxygen availability, altered carbon sources, and spatial pH gradients [40, 41]. To persist under these conditions, Mtb undergoes extensive metabolic and physiological adaptation that maintains cellular homeostasis while minimizing energy expenditure [42, 43]. These adaptive responses are increasingly recognized as important contributors to bacterial persistence and phenotypic drug tolerance.

Within this adaptive framework, CAs catalyze the rapid interconversion of CO_2_ and bicarbonate, thereby maintaining intracellular CO_2_/HCO_3_⁻homeostasis required for multiple metabolic processes [21, 43]. Under hypoxic conditions, respiratory activity decreases and central carbon metabolism is extensively reprogrammed, including changes in bicarbonate-dependent metabolic reactions [26, 41, 44]. Similarly, within the acidic phagolysosomal compartment of macrophages, mycobacteria must preserve intracellular pH and maintain enzymatic activity despite substantial environmental stress [41, 45]. Through the reversible hydration of CO₂, CAs contribute to intracellular pH buffering; whether this activity has a specific role in supporting mycobacterial adaptation to acid stress remains to be experimentally established. Although the established biochemical functions of CAs support these proposed physiological roles, their specific contribution to bacterial adaptation during antibiotic exposure remains insufficiently defined.

Beyond their role in pH homeostasis, CAs may facilitate the metabolic flexibility that enables mycobacteria to utilize alternative carbon sources during infection. Under nutrient-limited conditions, Mtb shifts from carbohydrate metabolism toward the utilization of host-derived lipids and fatty acids, requiring extensive metabolic reprogramming together with bicarbonate-dependent carboxylation reactions, including those catalyzed by acetyl-CoA carboxylase and propionyl-CoA carboxylase, which contribute to lipid biosynthesis and the metabolism of host-derived carbon substrates [35–46]. By maintaining intracellular bicarbonate availability, CAs could potentially support bicarbonate-dependent metabolic and biosynthetic processes during adaptation to nutrient limitation, although this relationship remains to be experimentally established. Because metabolic flexibility is a defining feature of persistent mycobacteria, CA-mediated bicarbonate homeostasis may be associated with metabolic processes that support persistent bacterial populations, although a direct role in persistence remains unestablished.

Stress-induced metabolic remodeling is frequently accompanied by reduced growth rates and the transition into non-replicating or slowly replicating physiological states that exhibit increased tolerance to environmental and antibiotic stress [45]. These adaptive states are characterized by altered energy metabolism, reduced biosynthetic activity, and extensive physiological remodeling [11, 16, 42]. Whether CAs actively regulate these transitions or simply support the metabolic processes required to sustain them remains an important unresolved question. Addressing this distinction will be important for determining whether CAs represent biomarkers of bacterial adaptation or mechanistic contributors to persistence and drug tolerance.

Collectively, these observations support the hypothesis that CAs may contribute to the mycobacterial metabolic stress-response network. Through their established roles in bicarbonate homeostasis and associated metabolic processes, CAs could potentially contribute to bacterial adaptation within hostile host environments, although this relationship remains to be experimentally established. Rather than directly conferring antibiotic tolerance, CAs may influence metabolic states associated with phenotypic drug tolerance. This perspective provides a conceptual framework for examining how metabolic homeostasis may be associated with bacterial persistence during TB infection, independently of classical resistance mechanisms.

## CAs and Antimicrobial Susceptibility

AMR in mycobacteria is primarily driven by genetic mechanisms, including target-site mutations, reduced drug permeability, enzymatic drug modification, and multidrug efflux systems [47, 48]. However, increasing evidence indicates that antibiotic efficacy is also influenced by the physiological and metabolic state of bacterial cells, independent of stable resistance-conferring mutations [49, 50]. Rather than acting as classical resistance determinants, CAs may indirectly influence antimicrobial susceptibility through their roles in bicarbonate homeostasis, intracellular pH, and central carbon metabolism, although this relationship remains to be experimentally established [26]. Consequently, CA-associated metabolic processes could potentially influence bacterial responses to antibiotic exposure without directly mediating genetic antimicrobial resistance, although this possibility remains to be experimentally established.

One hypothetical mechanism could involve an indirect relationship between CA-mediated metabolic homeostasis and the cellular bioenergetics required for energy-dependent resistance processes. Multidrug efflux pumps rely on either the proton motive force or ATP-dependent transport systems to actively export antibiotics from bacterial cells [51]. Given their roles in intracellular pH regulation and metabolic homeostasis, CAs could hypothetically influence the energetic conditions required for efflux activity. However, direct experimental evidence linking CA activity to efflux pump function in Mtb remains limited, and this proposed relationship should currently be regarded as hypothetical [26, 52].

CA activity may also be associated with regulatory pathways involved in mycobacterial adaptation. Inhibition of CA activity has been reported to affect the PhoPR regulon and the ESX-1 secretion system, resulting in attenuation of virulence [30]. In parallel, CO_2_ functions as an environmental signal regulating PhoPR-dependent pathways, highlighting the importance of CO_2_/HCO_3_⁻ homeostasis in mycobacterial physiology [31]. These observations suggest that CAs may be associated with broader metabolic and regulatory networks involved in environmental adaptation; whether these relationships influence responses to antibiotic stress remains to be experimentally established.

An additional mechanism by which CAs may affect antimicrobial susceptibility is through their influence on cell envelope metabolism. The lipid-rich mycobacterial cell envelope forms a major permeability barrier that restricts antibiotic entry [26, 30]. The biosynthesis and remodeling of several cell-envelope lipid components involve bicarbonate-dependent metabolic pathways [26]. Accordingly, disruption of CA-mediated bicarbonate homeostasis could potentially alter cell-envelope integrity and permeability, although this proposed relationship and its consequences for antibiotic susceptibility remain to be experimentally established. Experimental evidence supporting this mechanism, however, remains limited and warrants further investigation.

Experimental studies employing carbonic anhydrase inhibitors (CAIs) provide evidence that pharmacological inhibition of CA activity can impair mycobacterial growth and viability under specific experimental conditions [19]. Inhibition of CA activity has been associated with impaired bacterial growth and reduced viability under specific experimental conditions [22, 23, 53]. Although CA inhibition has demonstrated direct antimycobacterial activity in experimental studies [22, 24, 53], evidence that CAIs specifically enhance the activity of conventional antimycobacterial agents remains limited. These observations establish CA inhibition as a potential direct antimycobacterial strategy but do not demonstrate reversal of phenotypic drug tolerance or antibiotic resensitization. Testing the latter hypothesis will require specifically designed combination studies in which CA inhibition is evaluated together with conventional antibiotics in drug-tolerant bacterial populations. Such studies should include clinically relevant infection models, multiple antibiotic classes, and genetically defined CA mutants before definitive conclusions can be drawn [28, 54].

Overall, current evidence suggests that CAs should not be regarded as classical AMR determinants but rather as metabolic regulators that may influence bacterial susceptibility to antibiotics. By modulating bicarbonate homeostasis, intracellular pH, carbon metabolism, and associated regulatory pathways, CAs may influence physiological states potentially associated with altered antibiotic susceptibility and phenotypic drug tolerance. Defining these mechanistic relationships represents an important step toward evaluating whether CAs could serve as therapeutic targets, including their potential use in adjunctive treatment strategies.

## CAs and Drug Tolerance: A Metabolic Perspective

Drug tolerance in Mtb refers to the ability of a bacterial population to survive prolonged antibiotic exposure without acquiring heritable resistance-conferring mutations, whereas persistence refers to survival of a subpopulation of phenotypically tolerant cells [13, 55]. In contrast to antimicrobial resistance, which results from stable genetic alterations, drug tolerance represents a reversible physiological state associated with multiple adaptive processes, including metabolic remodeling [3, 11, 55]. During infection, a fraction of the bacterial population transitions into slowly replicating or non-replicating states that exhibit markedly reduced susceptibility to bactericidal antibiotics. Consequently, persister populations may contribute to prolonged treatment and disease relapse and may ultimately facilitate the emergence of genetically resistant strains [56, 57].

As discussed in the preceding sections, host-derived stresses trigger extensive metabolic remodeling that enables mycobacteria to survive under adverse conditions. Rather than considering CAs as isolated metabolic enzymes, an emerging view is that they may participate in maintaining the physiological state from which phenotypic drug tolerance develops. This hypothesis places bicarbonate homeostasis at the interface between environmental adaptation and bacterial persistence.

Drug-tolerant mycobacteria are characterized by coordinated reductions in energy metabolism, biosynthetic activity, and cellular replication while maintaining sufficient metabolic function to preserve viability. These physiological adaptations reduce the effectiveness of antibiotics whose bactericidal activity depends on active growth and metabolism [58, 59]. Maintenance of intracellular bicarbonate availability and pH homeostasis may contribute to cellular stability during these metabolically restricted states, although their specific importance for bacterial persistence remains to be experimentally established.

**Fig. 1:**
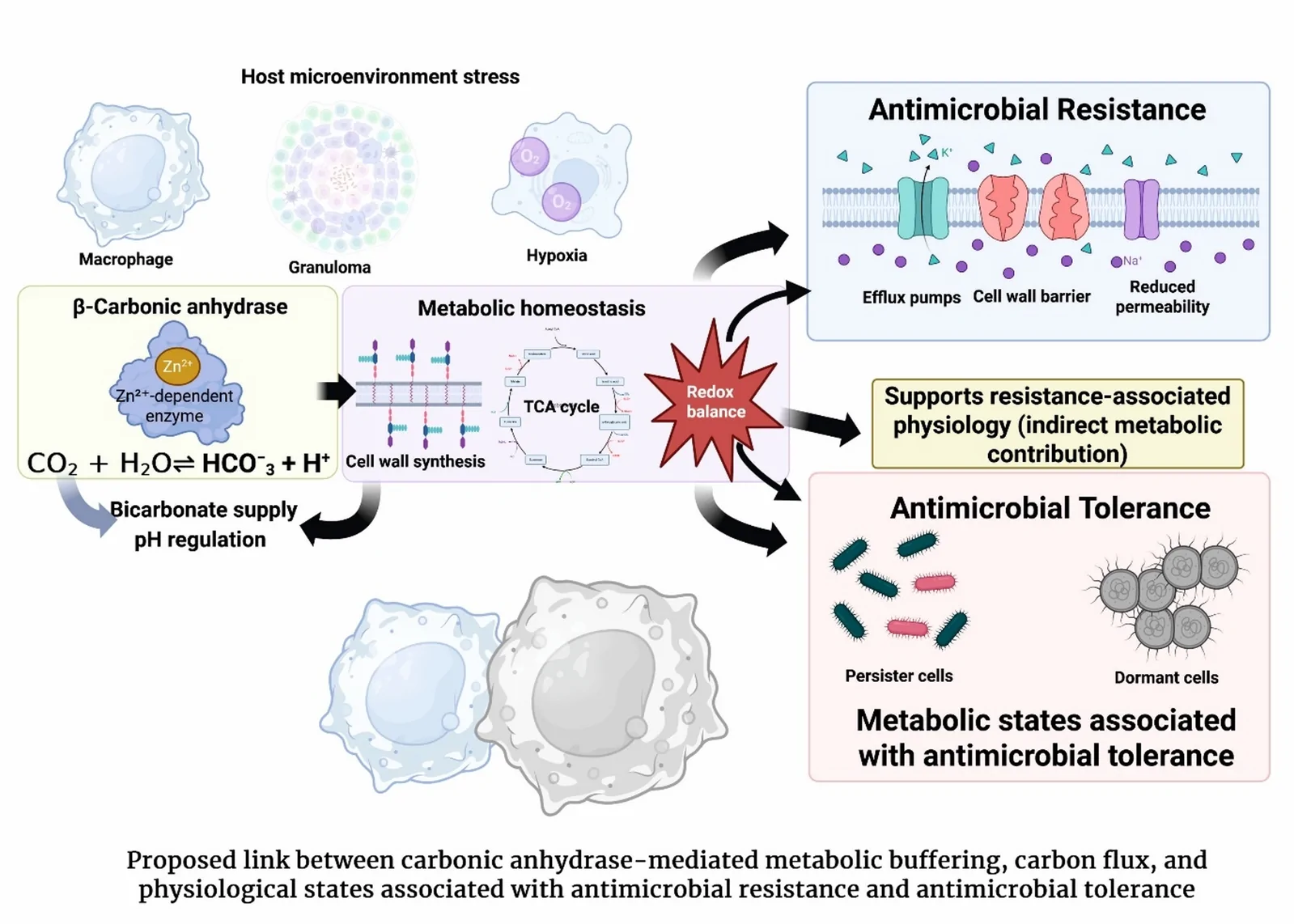
Proposed role of CAs in metabolic adaptation and phenotypic drug tolerance in mycobacteria. CAs, predominantly β-class enzymes in mycobacteria, catalyze the reversible hydration of carbon dioxide (CO₂) to bicarbonate (HCO₃⁻) (CO_2_ + H_2_O ⇌ HCO₃⁻ + H⁺), thereby contributing to intracellular pH regulation and bicarbonate supply. These processes support key metabolic pathways, including lipid biosynthesis, anaplerotic reactions, and cellular energy balance, which are essential for maintaining metabolic homeostasis. Through their role in regulating carbon flux and proton balance, CAs may indirectly influence bacterial susceptibility by supporting physiological processes such as energy-dependent transport, cell envelope integrity, and intracellular metabolic homeostasis. Importantly, CA-mediated metabolic buffering may facilitate the establishment and maintenance of drug-tolerant states characterized by reduced growth, persistence, and adaptation to stress conditions, such as hypoxia and nutrient limitation. This integrative model highlights CAs as candidate metabolic nodes linking environmental stress, intracellular homeostasis, altered antimicrobial susceptibility, and phenotypic drug tolerance in mycobacteria

Within this framework, CAs represent plausible contributors to the metabolic landscape associated with drug tolerance, although direct experimental evidence linking CA activity to tolerance phenotypes remains limited [19, 23, 29, 30]. By catalyzing the reversible interconversion of CO_2_ and bicarbonate, these enzymes regulate intracellular bicarbonate availability and contribute to pH homeostasis, both of which are closely linked to central metabolism [26]. Rather than directly inducing drug tolerance, CAs may support the metabolic stability required for bacteria to persist during prolonged antibiotic exposure. These interconnected metabolic processes are summarized in Fig. 1.

An additional consideration is the role of CAs in sustaining bicarbonate-dependent biosynthetic pathways during metabolic restriction. By supporting anaplerotic reactions, lipid metabolism, and other bicarbonate-dependent processes, CAs may contribute to the maintenance of essential cellular functions even when overall metabolic activity is substantially reduced [29, 31, 60]. Such metabolic buffering could potentially contribute to transitions between active growth and persistent physiological states, thereby influencing bacterial resilience during environmental and antibiotic stress. Although the contribution of individual CA isoforms remains poorly defined, this concept provides a mechanistic basis for future investigation. A summary of CA isoforms, their functional roles, and their potential contributions to drug tolerance is provided in Table 1.

Importantly, many bactericidal antibiotics require metabolically active cells to achieve maximal killing. Processes such as cell wall synthesis, deoxyribonucleic acid (DNA) replication, transcription, and energy production are all diminished in drug-tolerant populations [5, 16, 61]. Accordingly, enzymes that help maintain metabolic homeostasis without promoting rapid growth may indirectly reduce antibiotic efficacy by sustaining bacterial viability during treatment. Whether CAs contribute to this metabolic buffering remains an important unresolved question that warrants direct experimental investigation.

From a therapeutic perspective, targeting metabolic pathways that support tolerance-associated physiological states represents a potential strategy for improving antimicrobial efficacy. Inhibition of CAs could potentially disrupt metabolic homeostasis and alter physiological conditions associated with bacterial persistence; whether this can enhance the susceptibility of drug-tolerant mycobacteria to conventional antibiotics remains to be experimentally established [28, 55]. This concept could be therapeutically relevant because it aims to target drug-tolerant bacteria without relying solely on the discovery of new antibiotics. However, the specific contributions of individual CA isoforms to drug tolerance remain poorly defined, and additional mechanistic studies are required to establish causal relationships.

Overall, CAs represent a potential metabolic link between environmental adaptation and phenotypic drug tolerance in mycobacteria. By influencing bicarbonate homeostasis, intracellular pH regulation, and central carbon metabolism, these enzymes may contribute to the physiological states associated with bacterial survival during prolonged antibiotic exposure, rather than directly mediating antimicrobial resistance. Future studies combining genetic manipulation, metabolomics, infection models, and isoform-specific inhibition will be important to determine whether CAs function as mechanistic drivers of persistence or as metabolic facilitators of drug-tolerant states.

**Table 1:** Role of CAs in mycobacterial physiology and their potential links to antimicrobial resistance and drug tolerance

CA Isoform / System	Biological Function	Metabolic / Cellular Role	Impact on AMR / Drug Tolerance	Therapeutic Implications	Evidence	References
β-CA1 (CanA)	Catalyzes reversible CO₂/HCO₃⁻ interconversion	Contributes to bicarbonate homeostasis and associated metabolic processes; isoform-specific physiological role remains incompletely defined	May support metabolic adaptation under stress; potential role in tolerance-associated states remains hypothetical	Targeting may disrupt metabolic homeostasis; effects on antibiotic susceptibility require experimental validation	Genetic and biochemical evidence for CA function; direct evidence for drug tolerance lacking	[19, 20]
β-CA2 (CanB)	Catalyzes reversible CO₂/HCO₃⁻ interconversion	Contributes to bicarbonate homeostasis and associated metabolic processes; isoform-specific physiological role remains incompletely defined	May contribute to survival under hypoxia and nutrient limitation; potential contribution to persistence-associated physiological states	Potential drug target; inhibition may affect bacterial fitness	Gene manipulation and inhibitor studies for CA function; direct evidence for drug tolerance lacking	[19, 20, 62, 63]
β-CA3 (CanC)	Catalyzes reversible CO_2_ /HCO_3_⁻ interconversion	Contributes to bicarbonate homeostasis and associated metabolic processes; isoform-specific physiological role remains incompletely defined	May contribute to adaptation under stress conditions; potential link to tolerance remains hypothetical	Potential therapeutic target; role in combination strategies requires experimental validation.	Bioinformatic and functional evidence for CA properties; direct evidence for drug tolerance lacking	[23, 64]
γ-CA	Alternative CA system	May function under specific environmental conditions	Role not well defined; potential involvement in metabolic resilience remains hypothetical	Potential therapeutic target; requires further validation	Structural and enzymatic evidence for CA properties; role in drug tolerance remains unestablished	[21]
CA-mediated pH regulation	Maintains cytoplasmic pH balance	Contributes to buffering under acidic stress	May support adaptation under hostile environmental conditions; potential contribution to tolerance-associated physiological states remains unestablished	Inhibition may disrupt pH homeostasis and reduce survival	Biochemical and physiological evidence for pH homeostasis; direct evidence for drug tolerance lacking	[19]
Bicarbonate-dependent metabolism	Provides substrate for carboxylation reactions	Supports central carbon metabolism and lipid biosynthesis	May contribute to metabolic conditions associated with stress adaptation; a direct role in drug tolerance remains unestablished	Disruption may affect metabolic stability.	Metabolic studies; indirect evidence	[29, 65]
Redox homeostasis (hypothesized)	May contribute to NAD⁺/NADH balance (indirect).	May be indirectly linked to respiration and energy metabolism.	Redox balance is associated with persistence and tolerance states; a CA-specific relationship remains hypothetical	CA inhibition may influence redox balance indirectly	Systems biology and experimental evidence for redox metabolism; CA-specific relationship remains hypothetical	[26]
CA inhibition (pharmacological)	Inhibits CO_2_ hydration	Reduces bicarbonate availability and may disrupt pH homeostasis	May induce metabolic stress; effects on antibiotic susceptibility remain to be experimentally established.	Potential adjunct strategy with existing antibiotics; requires experimental validation	Inhibitor screening; zebrafish and in vitro models	[66, 67]

## Targeting Carbonic Anhydrases: Therapeutic Opportunities

CAs represent potential therapeutic targets because of their established biochemical role in CO_2_/HCO_3_⁻interconversion and their contribution to bicarbonate homeostasis and associated metabolic processes in Mtb [19, 20, 26, 67]. Unlike conventional antitubercular strategies that target established bacterial processes, targeting CAs may provide a strategy to perturb metabolic processes associated with bacterial adaptation to host-imposed stress. This concept is particularly relevant in the context of phenotypic drug tolerance, where targeting bacterial metabolism may complement conventional antimicrobial therapy.

**Fig. 2:**
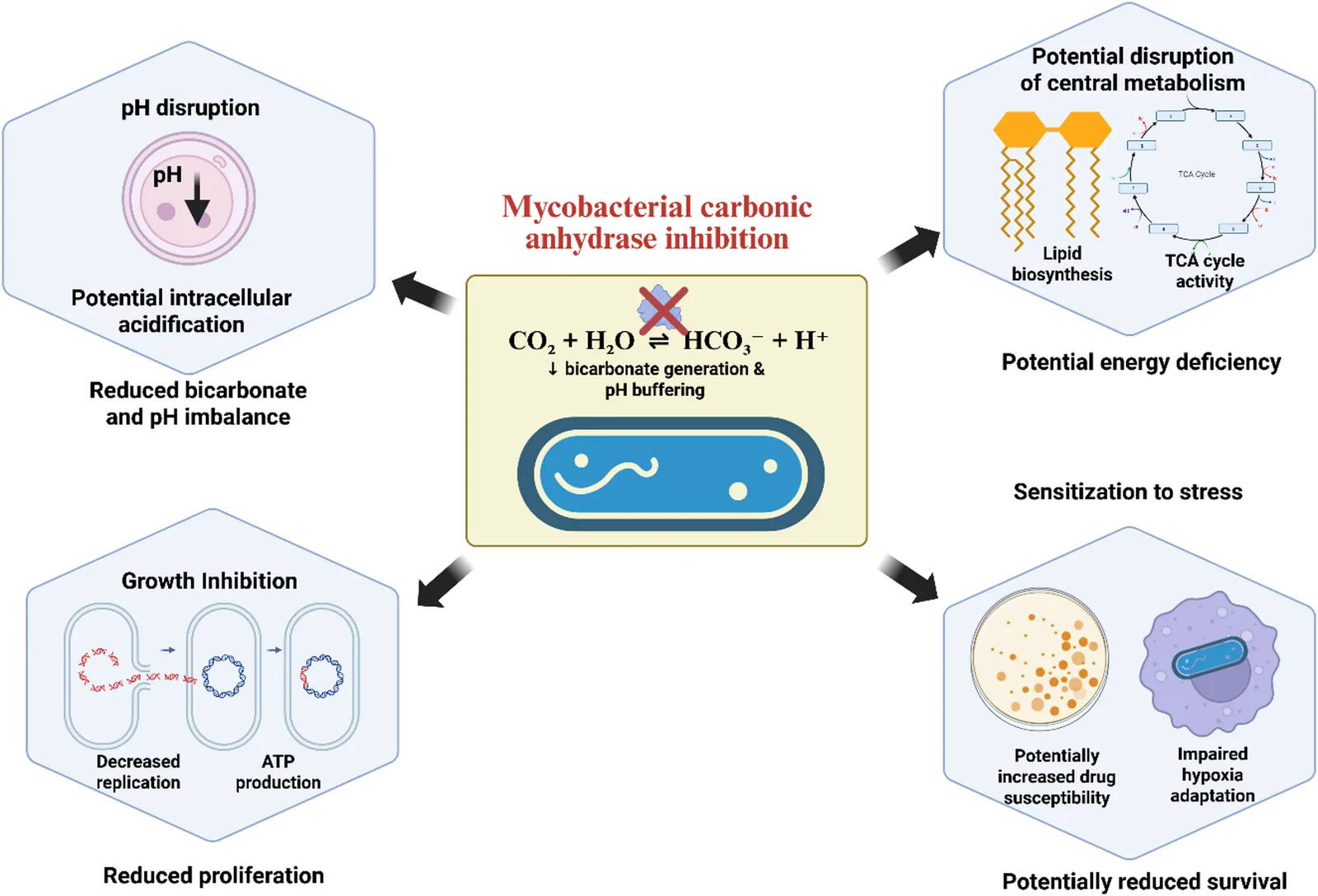
Proposed consequences of mycobacterial CA inhibition on cellular metabolism and drug response. Inhibition of mycobacterial CAs disrupts the reversible hydration of carbon dioxide, reducing bicarbonate (HCO_3_⁻) generation and potentially impairing intracellular pH homeostasis. Reduced bicarbonate availability may affect bicarbonate-dependent carboxylation reactions and thereby influence central carbon metabolism, including TCA-associated pathways and lipid biosynthesis. These metabolic perturbations are proposed to affect cellular energy balance and may contribute to reduced bacterial growth and proliferation. In parallel, disruption of CA-dependent metabolic homeostasis may increase susceptibility to environmental and antibiotic-induced stress and impair adaptation to hypoxic and other host-imposed conditions. However, direct experimental evidence connecting these downstream effects of CA inhibition to phenotypic drug tolerance remains limited. Thus, the pathways illustrated here represent a conceptual framework integrating established biochemical functions of CAs with proposed consequences for metabolic adaptation, antimicrobial susceptibility, and bacterial survival

The structural biology, inhibitor classes, and medicinal chemistry of mycobacterial CAs have been comprehensively reviewed elsewhere and are therefore only briefly summarized here [19–24, 67]. Several classes of CAIs, including sulfonamides, sulfamates, coumarins, and related chemical scaffolds, inhibit enzyme activity by coordinating the catalytic zinc ion or preventing substrate access to the active site [20, 24, 67, 68]. The representative mechanisms of CA inhibition and their potential therapeutic implications are summarized in Fig. 2. Importantly, the structural divergence between bacterial β-class CAs and human α-class enzymes provides opportunities for the development of selective inhibitors with reduced host toxicity.

Experimental studies provide important proof-of-concept for targeting carbonic anhydrases. Pharmacological inhibition of CA activity using ethoxzolamide disrupts the PhoPR regulon and the ESX-1 secretion system, resulting in attenuation of Mtb virulence [30]. Inhibition of CA activity has also been associated with impaired bacterial growth and altered metabolic activity under conditions requiring efficient CO₂/HCO₃⁻ homeostasis [22, 23]. Although these findings do not directly demonstrate reversal of drug tolerance, they establish that perturbation of bicarbonate metabolism produces measurable physiological consequences in mycobacteria.

Carbonic anhydrase inhibition may have therapeutic potential through two distinct, but not mutually exclusive, strategies. First, several CAIs have demonstrated direct antimycobacterial activity in vitro and in vivo, supporting the development of mycobacterial CAs as direct antimicrobial targets [22, 24, 53]. Second, CA inhibition could be explored as an adjunctive strategy in combination with conventional antitubercular agents. In this context, disruption of bicarbonate homeostasis and intracellular pH regulation may alter the physiological state of persistent bacteria, although its effects on antibiotic susceptibility remain to be experimentally established. However, direct antimycobacterial activity of a CAI should not be interpreted as evidence of antibiotic resensitization. Demonstrating a specific adjunctive or tolerance-reversing effect will require appropriately designed combination studies that distinguish direct CAI-mediated growth inhibition from enhancement of antibiotic activity against drug-tolerant populations.

Recent advances in computational drug discovery have accelerated the identification of novel CA inhibitors. Structure-based drug design, virtual screening, molecular docking, pharmacophore modeling, and molecular dynamics simulations have facilitated the discovery and optimization of isoform-selective inhibitors [19, 24]. These computational approaches should be viewed as complementary to experimental validation using biochemical assays, macrophage infection models, zebrafish infection models, and ultimately mammalian models that evaluate antimicrobial efficacy and host toxicity [69–72].

Despite these advances, several important challenges remain. Achieving isoform selectivity within the bacterial CA family, ensuring adequate penetration through the lipid-rich mycobacterial cell envelope, and demonstrating efficacy in physiologically relevant infection models continue to represent major barriers to clinical translation [19]. Furthermore, direct evidence linking inhibition of individual CA isoforms to reduced persistence or reversal of phenotypic drug tolerance is currently lacking. Addressing this knowledge gap will be important for future translational studies.

Overall, targeting CAs represents a potential metabolism-based therapeutic strategy for TB that warrants further experimental investigation. In addition to their potential as direct antimicrobial targets, CAs warrant investigation as targets for perturbing physiological adaptations associated with bacterial persistence during antibiotic treatment. Future therapeutic development should therefore focus not only on improving inhibitor potency and selectivity but also on determining whether CA inhibition can enhance antibiotic killing of drug-tolerant bacterial populations.

## Leveraging the *Mycobacterium marinum*–Zebrafish Model to Investigate CAs and Drug Tolerance

The *M. marinum*–zebrafish model provides a well-established in vivo system for investigating mycobacterial pathogenesis, host–pathogen interactions, and antimicrobial responses. Its ability to reproduce granuloma-like infection environments and permit real-time visualization of infection makes it particularly useful for future studies examining bacterial adaptation under host-imposed stresses [73–79]. In the context of this review, we therefore consider the model primarily as an experimental platform for testing, rather than supporting, the proposed relationship between CA-mediated metabolic adaptation and phenotypic drug tolerance.

The *M. marinum*–zebrafish model could be used to test the in vivo contribution of CAs to metabolic adaptation by combining genetically modified bacterial strains with pharmacological CA inhibition. Such studies could determine whether disruption of CA-mediated bicarbonate homeostasis alters bacterial fitness, persistence, or antimicrobial susceptibility within granuloma-like microenvironments, thereby directly testing the hypotheses proposed in this review.

The model could also support future evaluation of CAIs by enabling antimicrobial activity and host toxicity to be assessed within the same in vivo system, thereby helping prioritize compounds for subsequent validation in mammalian infection models [53, 63, 80–84].

Most importantly, the *M. marinum*–zebrafish system offers a future experimental platform for testing a key hypothesis of this review: whether disruption of CA-mediated bicarbonate homeostasis alters physiological states associated with persistence and phenotypic drug tolerance. This could be investigated by comparing wild-type and CA-deficient *M. marinum* strains, or by pharmacologically inhibiting individual CA isoforms, followed by antibiotic exposure in infected zebrafish [85]. Monitoring bacterial burden during treatment and regrowth following antibiotic withdrawal, with metabolic profiling where feasible, could help distinguish the direct effects on bacterial growth from the effects on persistence and drug tolerance.

Future studies combining isoform-specific CA mutants, pharmacological inhibition, metabolic profiling, and in vivo imaging in the *M. marinum*–zebrafish system could help determine whether CAs actively contribute to persistence and phenotypic drug tolerance or primarily support the metabolic state associated with these phenotypes.

## Future Perspectives and Concluding Remarks

Growing recognition of the role of metabolic adaptation in antimicrobial susceptibility and phenotypic drug tolerance has opened new avenues for therapeutic intervention in mycobacterial infections [48]. Within this framework, CAs represent potential therapeutic targets; however, their precise mechanistic contribution to bacterial persistence remains unclear. A major priority for future research will be to determine whether CAs actively regulate adaptive physiological transitions or primarily support the metabolic homeostasis required to sustain them. Resolving this distinction will be important for defining the therapeutic value of targeting these enzymes.

A key research priority is the integration of genetic, metabolic, and functional approaches to clarify the contribution of CAs to bacterial adaptation and survival during antibiotic exposure. Combining transcriptomics, metabolomics, proteomics, and targeted functional studies across complementary experimental systems—including macrophage infection models, zebrafish, and mammalian infection models—could provide a more comprehensive understanding of how CA-mediated processes influence the physiological states associated with drug tolerance [86]. Particular emphasis should be placed on isoform-specific genetic manipulation and metabolic profiling to establish causal, rather than associative, relationships between carbonic anhydrase activity and bacterial persistence.

From a therapeutic perspective, combination strategies targeting both classical AMR mechanisms and metabolic adaptation represent a potential direction for future research. The use of CAIs together with established antitubercular drugs could be investigated as a strategy to disrupt metabolic states associated with bacterial persistence; whether such combinations enhance antimicrobial efficacy remains to be experimentally established [19, 20]. Rather than replacing existing antibiotics, CA inhibition could be investigated as an adjunctive strategy, although its potential effects on treatment duration, relapse, and the emergence of genetically resistant populations remain to be experimentally established. However, the efficacy, selectivity, and safety of such approaches remain to be established in physiologically and clinically relevant models.

Advances in computational biology and artificial intelligence may facilitate the discovery and optimization of CAIs. Machine learning-guided virtual screening, structure-based drug design, molecular dynamics simulations, and integrative multi-omics analyses provide useful tools for identifying and prioritizing novel inhibitors for subsequent experimental validation [19]. Future progress, however, will depend equally on rigorous experimental validation that integrates structural biology with functional microbiology and infection biology.

Beyond CAs themselves, this review highlights a broader shift in TB research. Increasing attention is moving away from viewing AMR solely as a consequence of genetic mutations toward understanding how bacterial metabolism, environmental adaptation, and physiological plasticity influence treatment outcomes [87]. Within this emerging paradigm, CAs provide a useful framework for exploring how metabolic homeostasis contributes to persistence and phenotypic drug tolerance.

In conclusion, current evidence does not support CAs as classical AMR determinants. Rather, they should be viewed as metabolic enzymes whose potential contribution to physiological states associated with phenotypic drug tolerance remains to be experimentally established. This conceptual framework provides new opportunities for investigating the relationship between bicarbonate homeostasis, metabolic adaptation, and bacterial persistence while identifying metabolism-based therapeutic strategies that complement existing antitubercular chemotherapy. Future interdisciplinary studies integrating microbiology, structural biology, metabolomics, computational modeling, and in vivo infection models will be important for determining whether targeting CAs can meaningfully enhance TB treatment and improve clinical outcomes.

## Data Availability

No datasets were generated or analysed during the current study.
